# Self-medication for infants with colic in Lagos, Nigeria

**DOI:** 10.1186/1471-2431-9-9

**Published:** 2009-02-04

**Authors:** Kazeem A Oshikoya, Idowu O Senbanjo, Olisamedua F Njokanma

**Affiliations:** 1 Pharmacology Department, Lagos State University College of Medicine, P.M.B 21266, Ikeja, Lagos, Nigeria; 2 Paediatrics and Child Health, Lagos State University Teaching Hospital, Ikeja, Lagos, Nigeria; 3 Academic Division of Child Health, University of Nottingham, The Medical School, Derbyshire Children's Hospital, Uttoxeter Road, Derby, DE22 3DT, UK

## Abstract

**Background:**

Infantile colic is a self-limiting condition that is distributed worldwide. It is often misdiagnosed as an organic disease for which an infant is admitted to the hospital. Many studies have described the aetiopathogenesis, pharmacologic and non-pharmacologic management of colic but none has evaluated self-medication for infants with colic. The aim of this study was therefore to determine the knowledge of Nigerian mothers about colic, their home-based management, extent of self-medication for the infants with colic and the types of medicines involved.

**Methods:**

It is a prospective study conducted at the vaccination clinics of 20 primary health care centres, each from different Local Government Areas in Lagos, Nigeria. Eight hundred mothers that brought their infants for vaccination between April and September, 2006 were interviewed with open-and close-ended questionnaire.

**Results:**

Six hundred and eighty three (85.4%) mothers claimed they had a good knowledge of colic. Incessant and excessive cry was the main clinical feature of colic identified by 430(62.9%) mothers. Three hundred and seventy eight (67.7%) infants were treated by self-medication, 157 (28.1%) sought medical intervention and 17 (3.1%) were treated at a traditional birth attendant home. Herbal medicines constituted 51.8% of the self-medicated medicines, of which 48 (26.2%) were "Ororo Ogiri". Nospamin^® ^(49.5%) and Gripe water^® ^(43.0%) were the two frequently prescribed and self-medicated medicines for infants with colic.

**Conclusion:**

Nigerian mothers are deficient in their knowledge of colic. Self-medication was the most frequently used home-based intervention. Health education would appear necessary to improve parental management of this self-limiting condition.

## Background

Infantile colic is a syndrome characterised by excessive, unexplained paroxysmal crying in an otherwise healthy baby [1-3]. The crying typically starts in the first few weeks of life and spontaneously resolves between 3–5 months [1-4]. Excessive crying is defined as crying that lasts more than three hours per day and more than three days per week for at least 3 weeks [2-5]. The cry has been typically described as a high-pitched scream, occurring mainly in the late afternoon or evening but may occur at any time. Characteristically, the infants' knees are drawn up to the abdomen, face is flushed, fists are clenched, flatus is expelled, the facies is "pained" and there is minimal response to attempts at soothing [1].

Colic affects 5% to 25% of infants throughout the world, depending on the study design, definition of colic and method of data collection [6,7]. It is derived from the Greek word *colon*, reflecting that it involved some bowel disturbances [7]. Previous studies have attributed colic to painful intestinal contractions, lactose intolerance, presence of gas in the gut, and parental misinterpretation of normal crying [2]. However, in recent times, it was hypothesised that infantile colic may have a medical or behavioural cause [3]. The medical hypotheses include food hypersensitivity or allergy [8,9] and immaturity of gut function or gut dymotility [3,10,11]. The behavioural hypotheses include inadequate maternal-infant interaction [12], maternal anxiety [5] and difficult infant temperament [13]. Other recent hypotheses that are being investigated are hormone alterations [11,14] and maternal smoking [3]. The role of gut microflora in the aetiopathogenesis of colic has been reported too [15-17]. The linking of colic to organic causes has changed its management interventions which include the use of wider range of pharmacological agents such as antispasmodic [18,19], defoaming agents [3], gut hormone antagonists [11,14], herbal medicines [19-21] and probiotics [6,22].

Both behavioural and pharmacological interventions have been studied over the years in the management of colic and have demonstrated a significant improvement in the symptoms [3,13,23]. However, only a few of these interventions have been confirmed by randomised clinical trials [3]. Infantile colic usually has a favourable course and outcome; even without treatment. Most infants are free of the symptoms by the age of 4–5 months [3]. However, most parents are faced with tremendous stress and are unlikely to tolerate the stress until the symptoms completely disappear without medical intervention; therefore medical help is either sought [24-26] or self- medication practiced by the parents [27]. Most doctors and nurses believe something has to be done to assist parents of infants with colic because of the stress [3,23]. The role of physicians in the management of colic is to exclude serious organic causes of inconsolable cry and offer a balanced advice on treatments [28]. At present, behavioural management, supportive counselling and parental reassurance are the mainstays of treatment [1,13,28]. Other non-pharmacological interventions such as feeding changes [29,30] and oral administration of sucrose solution [23] have been found useful in the management of colic.

The use of herbal medicines for common childhood illnesses has been reported in Nigeria [31,32] but its use in the management of colic has not been explored. About 85% of Nigerians are known to use and consult traditional medicine for healthcare, social and psychological benefits because of poverty and disillusionment with conventional medical care [33]. Only a few of the herbal medicines in circulation in Nigeria are registered by the National Agency for Food and Drug Agency and Control (NAFDAC) [34]. The importance of traditional medicine in Nigerian healthcare has been recognised by the national government who set up a high profile committee to develop, promote and commercialise traditional medicine products [35]. Efforts have also been made by the government to preserve indigenous Nigerian medical knowledge by boosting research into traditional medicine [35].

Self-medication is a global problem and is associated with adverse drug reactions [36], treatment failure [37] and resistance to antibiotics and antimalarials [38]. A 47.6% prevalence of self-medication has been reported among infants in Lagos, Nigeria. Abdominal pains, constipation, fever and cough are the most common symptoms of infants that are frequently treated by self-medication in Nigeria [31,32]. Previous studies that evaluated self-medication among infants in developing countries did not evaluate infants with colic [30,31,39]. In 1973, *Buchanan *first reported self-medication for colic in an urban black population in South Africa [27]. A major limitation of the study was that self-medication was assessed in a general population predominated by adults. Since pharmacological intervention remains one of the management strategies of colic, it is appropriate to assess the extent of self-medication in the management of this condition. This study was therefore aimed to determine the knowledge of Nigerian mothers about colic, their home-based management, extent of self-medication for infants with colic and the types of medicines involved.

## Methods

Lagos is the smallest state but the most populous city in Nigeria with an estimated population of about 15 million inhabitants as of 1991 national census. It is divided into five divisions and twenty Local Government Areas (LGAs). There is at least a minimum of three Primary Health Care (PHC) centres in each LGA. One PHC was selected randomly from each LGA, thus a total of twenty PHC centres were used for data collection.

The study was conducted prospectively between April and September, 2006. Mothers who brought their infants for vaccination were randomly selected (40 from each PHC) and interviewed with a structured questionnaire. A total of 800 mothers were interviewed. The questionnaire was designed to obtain the demographics of both the mothers and their infants, and to assess their knowledge about infantile colic and how it was managed. Among those mothers that managed their infant's colic at home, the types of intervention used were sought. If pharmacological intervention was used, the types of medicine used and their sources were also sought. Similarly, among those infants managed for colic in hospitals, information on the medical advice given to the mothers and the medicines prescribed were also obtained with the questionnaire. The questionnaire was both open- and close-ended, developed from literature review on infantile colic [1-20] and from the opinion of mothers interviewed in a pilot study. In addition to literature review, previous studies that evaluated self-medication in Nigerian infants [31-33] and the opinion of community pharmacies and traditional herbal medicine practitioners were used to develop part of the questionnaire that focused on home management of colic. The interview was conducted by matrons in charge of the PHC, who have been trained for the study. The interview lasted about 30 minutes and was conducted in a consulting room. A mother was interviewed at a time to allow privacy. Only the mothers with a healthy infant and consented to participate in the study were interviewed.

A pilot study was carried out at one of the excluded PHC centres to enable us correct all the identified problems in the questionnaire. Most of the mothers suggested additional medicines and herbal medicine products that were not included on the questionnaire which they have used to treat infantile colic.

The data collected were analysed using SPSS version 13. Statistical analysis was by Fischer exact and Chi-square tests at a significant value of *P *< 0.05.

## Results

### Demography of the mothers and their infants

Most of the mothers (36.1%) were in the age group 21–25 years (range 15 to 40 years). The mothers' level of education ranged from illiteracy (2.5%) to university education (25.0%). Two hundred and twenty (36.3%) mothers were traders, 212 (26.5%) professionals and others were unemployed, students or artisans. The age of the infants ranged from one day to12 months. A majority of the infants (51.63%) were in the age group of one to 6 months. Their mean age was 4.9 ± 3.3 months. Four hundred and thirteen (51.6%) of the infants were female and 387 (48.4%) were male. Seven hundred and twenty (90.0%) infants were exclusively breastfed, 63 (7.9%) were partly breast fed and partly fed with formula feed, and 17 (2.1%) were exclusively fed with formula feed.

### Knowledge of mothers about infantile colic

Six hundred and eighty three (85.4%) mothers claimed they had a good knowledge of colic. A majority of the mothers (59.6%) were informed about colic by their parents, grand parents and personal experience from their previous children with colic. Other sources of information about colic are summarised in Table 1.

**Table 1:** Sources of information about infantile colic

Source	Frequency (n = 683)	Percentage (%)
Parents/grand parents	190	27.8
Experience from previous child/children	163	23.9
Experience from previous child/children + Parents/grand parents	61	8.9
In-laws	50	7.3
Parents/grand parents + in-laws	41	6.0
Friends	23	3.4
Traditional birth attendants	22	3.2
Friends + parents/grand parents	20	2.9
Experience from previous child/children + Friends	19	2.8
Hospital	15	2.2
Nurse	12	1.8
Health centre	11	1.6
Neighbours	11	1.6
Friends + relations + parents/grand parents	10	1.5
Friends + relations	9	1.3
Reading from books/TV/Radio programmes	9	1.3
Relations	9	1.3
Internet	8	1.2

The features of colic, as perceived by the mothers, are presented in Table 2. Only incessant and excessive cry was correctly identified by 430 (62.9%) mothers as a main feature of infantile colic. There was a significant increase in the proportion of mothers with higher education who correctly identified incessant and excessive cry as a main feature of colic when compared with mothers with secondary and primary education (*P *= 0.023). However, mothers between 30–40 years and those younger did not differ significantly in their knowledge of incessant and excessive cry as a main feature of colic (*P *= 0.062). Refusal of feeds, inconsolable cry and abdominal pains were the three most frequent incorrect features of colic wrongly identified as correct features.

**Table 2:** Signs and symptoms of colic identified by the mothers

Features	Yes (%)	No (%)
**Correct features**		
Incessant and excessive cry	62.6	37.4
Irritability	21.9	78.1
Passage of flatus	17.6	82.4
Intermittent cry	13.2	86.8
Healthy state of the infant	8.8	91.2
Insomnia	4.4	95.6
**Incorrect features**		
Refusal of feeds	36.6	63.4
Inconsolable cry	29.3	70.7
Abdominal pains	17.6	82.4
Fever	7.3	92.7
All day cry	7.2	92.8
Passage of loose stool	3.1	96.9

N = 683 (total number of mothers that were knowledgeable of infantile coilc), n = number of mothers that responded to each of the questions, % response = n/N

Infantile colic related cry was correctly defined according to *Wessel et al *criteria by 423 (61.9%) mothers. Five hundred and sixty five (82.7%) mothers identified colic onset as one week of life, 417 (61.1%) mothers believed colic could last up to 5 months of life and 143 (20.9%) mothers believed that colic has a cause. Highly educated mothers were able to associate colic to a cause more than mothers with secondary and primary education (101/296 vs 42/367, *P *< 0.05), but the proportion of mothers between 30–40 years did not differ significantly from younger mothers that understood that colic has a cause (*P *= 0.058). Table 3 shows the list of the causes of colic as perceived by the mothers. Abdominal cramps with hyper-peristalsis (60, 39.8%) were the most frequently identified correct cause of colic. Contrarily, 34 (23.8%) mothers wrongly identified worm, breast milk allergy and brain problem as causes of colic.

**Table 3:** Perceived causes of infantile colic

Causes	Yes (%)	No (%)
**Hypothesised (correct) causes**		
Abdominal cramps with hyper-peristalsis	39.8	60.2
Presence of gas in the intestine	16.1	83.9
Gastroesophageal reflux	11.9	88.1
Allergy to formula milk	4.2	95.8
Attachment to the mother	4.2	95.8
**Non-hypothesised (incorrect) causes**		
Worm	11.9	88.1
Allergy to breast milk	7.7	92.3
Brain problem	4.2	95.8

N = 143 (total number of mothers that perceived colic has a cause), n = number of mothers that responded to each of the questions, % response = n/N

Of the 683 mothers who claimed good knowledge of colic, 201 (36%) responded that their infants were currently experiencing colic. Colic was experienced at a mean age of 17.4 ± 1.0 days (range of 4 days to 2 months). One hundred and seventy (42.5%) infants with colic have older siblings that had experienced colic. The older siblings experienced colic at a mean age of 22.72 ± 1.18 days (range 4 days to 3 months) which was significantly higher than the mean age (17.4 ± 1.0 days) of the current infants with colic (P < 0.05).

### Mothers' management of colic

Of the 558 infants that experienced colic, 378 (67.7%) were treated at home by their mothers, 157 (28.1%) sought medical intervention in a hospital, 17 (3.1%) were treated by traditional herbal medicine practitioners; the remaining six were not given any treatment. There were no significant differences in the proportion of mothers with higher education and those with secondary and primary education, mothers between 30–40 years and those younger who employed home-based and hospital-based interventions, respectively, in the management of their infants' colic (*P *> 0.05). However, mothers who were professionals tend to use home-based interventions more than those who were traders (*P *= 0.012) for colic management. Contrarily, mothers who were traders sought hospital- based interventions more than those who were professionals (*P *= 0.023).

#### Self-medication

Of the 378 infants treated at home by the mothers, 353 (93.4%) were treated by self-medication. Levels of education and age of the mothers appear not to have a significant influence on the extent of self-medication for infants with colic (*P *= 0.073 and *P *= 0.055, respectively). Table 4 shows the list of self-medicated medicines used to treat infantile colic by mothers. Herbal medicines were the most frequently used medicines (51.8%), of which 48 (26.2%) were "Ororo Ogiri"; 22 (12.1%) were a mixture of *Allium asalonicum L*. (Onion) leaves,*Syzygium aromaticum L*. seeds and *Parinari *spp. seeds soaked in water; 21 (11.5%) were *Allium asalonicum L*. leaves only soaked in water; 13 (7.1%) were a mixture of naphthalene tablets, *Allium sativum L*.(Garlic) and *Allium asalonicum L*. leaves soaked in water; and 11 (6.0%) were *Allium sativum L*. only soaked in water. The components of the remaining 68 herbal medicines could not be ascertained by the mothers.

**Table 4:** List of self-medicated medicines used to treat infantile colic

Drugs	Frequency (n = 353)	Percentage (%)
Herbal medicines	183	51.8
Nospamin^®^	125	35.4
Gripe water^®^	106	30.0
Bonababe^®^	19	5.4
Piccan^®^	7	2.0
Kidcare^®^	4	1.1
Teething powder^®^	4	1.1
'Gbomoro'^®^	3	0.8
Paracetamol	3	0.8
Ascorbic acid	3	0.8
Ampicillin/cloxacillin	3	0.8

Multiple dugs used was observed here with an average drug used per patient of 1.3

Nospamin^®^: Homatropine methylbromide (an anticholinergic).

Gripe water^®^: Contains *Terpeneless dill *seed oil, sodium bicarbonate, ginger tincture and alcohol 0.221 ml.

Bonababe^®^: Contains paracetamol, diphenhydramine and concentrated dill water.

Piccan^®^: Contains paracetamol and diphenhydramine

Kidcare^®^: contains chloroquine.

Teething powder^®^: Contains tincture of matricaria and lactose.

'Gbomoro'^®^: Contains chloroquine.

The remaining self-medicated medicines were over-the counter medicines, except homatropine methylbromide [Nospamin^®^] (125, 35.4%) and ampicillin/cloxacillin (3). Most of the medicines were used either in combination with herbal medicines or in combination with other medicines.

#### Chiropractic intervention

In addition to parental self-medication, 120 (31.8%) infants were managed by massaging their abdomen with anointing oil or herbal mixtures. Mothers placing hands on the abdomen of their infants with colic or laying the infants on their abdomen (56.4%) and applying hot water bottle to the colicky abdomen (21.8%) were the other chiropractic interventions practised by the mothers.

#### Psychosocial intervention

One or more psychosocial interventions were adopted by 133 (35.2%) mothers, either alone or in combination with self-medication, in the management of colic. The psychosocial interventions included early response to the child while crying (24.8%), giving a pacifier to suck (18.1%), giving a gentle soothing motion (10.5%), avoidance of over-stimulation (9.8%) and carrying the infant in a carrier or a stroller (9.0%) to move around.

#### Combined non-pharmacological interventions

Thirty of the 35 infants with colic that were managed at home without self-medication were managed psychosocially and by massaging their abdomen with anointing oil or herbal mixtures. The rest were managed either psychosocially or by giving only abdominal massage.

### Hospital-based interventions for infantile colic

Amongst the 157 mothers that sought medical treatment for their infants' colic, 77 (49.0%) were treated only once and 68 (43.3%) were treated twice by doctors. Most of these infants (59.3%) were prescribed medicines. Thirty nine (24.8%) mothers were counselled about the cause and course of colic and the rest of the infants (15.9%) were investigated for the cause of abdominal pain. Nospamin^® ^(49.5%), Gripe water^® ^(43.0%), Piccan^® ^(12.9%), Erythromycin (10.8%), and Abidec^® ^(9.7%) were the medicines prescribed by doctors, either alone or in combination, for the treatment of colic.

## Discussion

Colic is a problem of infants with a high incidence reported worldwide [6,7]. An incidence rate of 36% was obtained in this study. This rate is slightly higher than the 5% to 30% previously reported in some studies [6,7,40-42] but falls in-between the 35% to 40% reported in other studies [43,44]. The differences in the incidence rates may be due to variations in the definition of colic and of differences in study design, method of data collection and population size between different studies [6,7]. In the present study, only the mothers that brought their infants for vaccination were interviewed, therefore, the reported incidence rate of colic may not represent the true incidence in Lagos.

Most of the mothers (85.4%) claimed that they were knowledgeable of the cause and course of colic. This was demonstrated by the ability of 82.7% of the mothers to correctly identify onset of colic as one to two weeks of life and 61.1% of the mothers being able to identify its complete disappearance by 5 months of life. The high level of education of the mothers may explain their good knowledge of colic. Colic has been reported to be more common in the first-born child and infants whose siblings had suffered colic [40]. Successful management of colic by grand mothers and mothers in their previous infants with colic would have earned them the knowledge and experience of colic. Therefore information from grandparents and experienced mothers is likely to be reliable in the management of colic. Unfortunately, the most reliable sources of information about colic (hospital, health centres, nurses, TV/radio and internet) were not sought by the majority of the mothers (Table 1). Therefore much publicity about colic, targeted at mothers, would appear necessary in Lagos. This can be achieved by health education talk in the vaccination and other paediatric clinics, and using posters and TV/radio jingles.

Over 60% of the mothers were able to define colic according to *Wessel's *criteria [45]. Incessant and excessive cry has been reported as a main feature of colic [2] and this was correctly identified by 62.9% of the mothers. The fact that a significant proportion of these mothers were highly educated shows that health education about colic at the vaccination clinic would likely be embraced by the mothers. However, other associated symptoms of colic that were not correctly identified by the mothers and the incorrect symptoms that were considered features of colic (Table 2) may lead to delay in seeking hospital care for serious illnesses that may mimic colic in infants. The incorrect features identified by the mothers such as refusal of feeds, inconsolable cry, abdominal pain, crying all day, fever and passage of loose stool are presenting features of malaria, sepsis or other life-threatening infectious diseases in infants [46]. Delay in their treatment could result in early infant morbidity and mortality.

Home-based management of colic was a common practice by the mothers. This finding was supported by the 67.7% of the mothers who treated their infants with colic without consulting with doctors. Parent reassurance, empathy, support and asking the mothers to take time-out when their infants were crying are home-based nursing interventions used to reduce parenting stress [13]. Other studies have shown that abdominal massage [23], administration of sucrose solution [23], and use of herbal tea [18,19] or hydrolysed formula [17] were equally very effective in reducing the duration of crying in infants with colic. These interventions are easy to apply and practicable at home by families without necessarily seeking the help of a doctor. However, parents must be able to properly identify symptoms of colic before adopting home-based interventions.

Self-medication and use of herbal medicines were the most practiced home-based interventions in this study unlike the non-pharmacological interventions recommended in other studies [13,23]. The 51.8% of mothers using home-based interventions were involved in the use of herbal medicines for their infants with colic. This high prevalence of herbal medicine use may be explained by the recognition given to traditional medicine in Nigerian healthcare by the Federal Government [35]. The use of herbal tea in the treatment of infantile colic has been reported in the developed countries [13,19,20] but the herbal medicines used by the infants in this study are quite different. Lagos is a metropolitan area, comprising of people from different tribes and cultures, therefore the population of mothers that participated in this study represent different cultures and tribes in Nigeria. The use of herbal medicines for infants with colic may therefore be considered a common practice among mothers of different culture and tribes in Nigeria but the types of herbal medicine used may likely differ. "Ororo Ogiri" (26.2%) was the mostly used herbal medicine. It is derived from putrefied *Cucumeropsis mannii *(melon) seeds which are used as a local food seasoning amongst the Yoruba tribe in Nigeria. When dissolved in water and drank by adults, it relieves indigestion by causing excessive farting and flatulence. Its use in the treatment of infantile colic was based on the hypothesis that it removes the excess intraluminal gas in the infant by causing flatulence. The safety of the herbal medicines in infants would necessitate their pharmacological and toxicological studies. We have earlier reported hepatic encephalopathy and death from the use of herbal preparations containing naphthalene tablets [36], therefore the 7.1% infants that were treated with a mixture of naphthalene tablets, *Allium sativum L*. and *Allium asalonicum L*. leaves soaked in water are at risk of such severe adverse reactions.

Infantile colic is self-limiting and has a favourable clinical course without treatment [2,47,48]. Although serious somatic problems are absent in most cases, yet doctors and nurses provide both medical and behavioural interventions to allay the stress posed by colic on the parents [47]. It is therefore not surprising to see 59.3% of the infants that were seen by doctors for colic prescribed medicines. It is interesting to know that 24.8% mothers were counselled about the cause and course of colic and 15.9% infants investigated for the cause of abdominal pain. Even though these percentages were low, they indicate that rational drug use can be achieved in the management of colic in Lagos. Nospamin^® ^is homatroprine methylbromide (an anticholinergic) which was prescribed to 49.5% infants and used in 35.4% infants without prescription. The use of anticholinergics for infantile colic as prescribed and non-prescribed medicines has been reported [18,42,49]. Evidence-based medicine has shown a clear benefit of these drugs in the treatment of excessive crying in infants [47]. However, their reported side effects in infants are of great concern [50] which may limit their use. There is much anecdotal evidence extolling the benefit of gripe water^® ^for colic but no formal evaluation of this medicine has been undertaken [51]. Gripe water^® ^has been in use for treating colic over 100 years ago [51]. This may explain the 43.0% doctors that prescribed the medicine and the 30.0% of mothers that were involved in its self-medication. It has been hypothesised that the alcohol content of gripe water^® ^provides a soothing effect [52], the bicarbonate provides a neutralizing effect to the gastric acid [51], and the carminative in the plant extract causes the soothing of the infant in the presence of excess gas in the lumen that may cause pain [53]. *Allium sativum L*. has been reported to produce flatulence in man [54] and *Syzygium aromaticum L*. is known to contain clove-oil as one of its constituents which is used as carminative [54]. The use of fennel oil similar to the clove-oil has been reported to be helpful in colic management. These treatments are however not entirely harmless [55], therefore proper dose has to be scientifically determined.

Laying the infant on the abdomen (56.4%), early response to the child while crying (24.8%), giving the infant a gentle soothing motion (10.5%) and avoidance of over-stimulation (9.8%) observed in this study are acceptable psychosocial methods of managing infantile colic which have been widely reported [1,22,47,56,57]. However, applying hot water bottle to the abdomen of the infant, as practiced by 21.8% mothers, has been reported to relieve rectal spasm to aid easier passage of flatus [58]. Although parents must be cautioned about the use of this method as they stand the risk of causing burn to the infants' abdomen.

The findings from this study may not be comparable to the general population since only a particular population of mothers were studied. The findings may be different if mothers attending other paediatric clinics were studied. This is however one of the limitations of the study.

## Conclusion

Nigerian mothers are deficient in their knowledge of cause and course of infantile colic. Self-medication was the most frequently used home-based interventions for infantile colic and was predominated by traditional herbal medicines. Nigerian mothers would need to be educated about colic through health education at paediatric and vaccination clinics, public health campaign and interdisciplinary team approach. Efficacy and toxicity of the traditional herbal medicines need to be established scientifically for their safe use for children. Government should strengthen the policy on sales and use of prescribed medicines in children without prescription so as to promote rational use of medicine in infants with colic.

## Abbreviations

NAFDAC: National Agency for Food and Drug Agency and Control; LGA: Local Government Authority; PHC: Primary Health Care.

## Competing interests

The authors declare that they have no competing interests.

## Authors' contributions

KAO conceived the study, designed the study and questionnaire, analysed the data, and drafted the manuscript. IOS critically reviewed the statistical analysis and participated in drafting the manuscript. FON participated in the design of the questionnaire and critically reviewed the manuscript.

## Pre-publication history

The pre-publication history for this paper can be accessed here:


